# An unbiased kinship estimation method for genetic data analysis

**DOI:** 10.1186/s12859-022-05082-2

**Published:** 2022-12-06

**Authors:** Wei Jiang, Xiangyu Zhang, Siting Li, Shuang Song, Hongyu Zhao

**Affiliations:** 1 Department of Biostatistics, School of Public Health, Yale University, New Haven, USA; 2 Department of Biomedical Data Science, Geisel School of Medicine, Dartmouth College, Hanover, USA; 3 Center for Statistical Science, Tsinghua University, Beijing, China; 4 Department of Industrial Engineering, Tsinghua University, Beijing, China

**Keywords:** Kinship estimation, Genomic relationship matrix, Unbiasedness

## Abstract

Accurate estimate of relatedness is important for genetic data analyses, such as heritability estimation and association mapping based on data collected from genome-wide association studies. Inaccurate relatedness estimates may lead to biased heritability estimations and spurious associations. Individual-level genotype data are often used to estimate kinship coefficient between individuals. The commonly used sample correlation-based genomic relationship matrix (scGRM) method estimates kinship coefficient by calculating the average sample correlation coefficient among all single nucleotide polymorphisms (SNPs), where the observed allele frequencies are used to calculate both the expectations and variances of genotypes. Although this method is widely used, a substantial proportion of estimated kinship coefficients are negative, which are difficult to interpret. In this paper, through mathematical derivation, we show that there indeed exists bias in the estimated kinship coefficient using the scGRM method when the observed allele frequencies are regarded as true frequencies. This leads to negative bias for the average estimate of kinship among all individuals, which explains the estimated negative kinship coefficients. Based on this observation, we propose an unbiased estimation method, UKin, which can reduce kinship estimation bias. We justify our improved method with rigorous mathematical proof. We have conducted simulations as well as two real data analyses to compare UKin with scGRM and three other kinship estimating methods: rGRM, tsGRM, and KING. Our results demonstrate that both bias and root mean square error in kinship coefficient estimation could be reduced by using UKin. We further investigated the performance of UKin, KING, and three GRM-based methods in calculating the SNP-based heritability, and show that UKin can improve estimation accuracy for heritability regardless of the scale of SNP panel.

## Introduction

Accurate estimation of relatedness among individuals is important in genetic data analysis. For example, in both population-based and family-based genome-wide association studies (GWAS) with uncertain relationships among study subjects, it is critical to appropriately account for cryptic relatedness because incorrect estimates can decrease power and inflate false positive rates of association tests [1–3]. It has been demonstrated that proper consideration of genetic relatedness can also benefit estimation of trait heritability based on GWAS data in the presence of pedigree structures [4, 5]. Several methods have been proposed to adjust for relatedness in large-scale human genetic association studies, such as introducing a genomic relationship matrix (GRM) as an augment into well-developed linear mixed model (LMM) [6–8]. For example, genome-wide complex trait analysis (GCTA) software models the GRM in the relationship between variance of phenotypes and the component explained by SNPs, and estimates heritability by the restricted maximum likelihood (REML) approach [4]. The GRM can also be incorporated to improve the performance of polygenic risk prediction, such as the genomic best linear unbiased prediction (gBLUP) method [9].

In order to adjust for cryptic relatedness in genetic studies like association mapping and heritability estimation, individual-level genotype data are often used to estimate pairwise kinship coefficients, which is defined as the probability that two homologous alleles drawn from each of two individuals are identical by descent (IBD). The methods can be mainly divided into likelihood estimators and method-of-moments estimators. The likelihood methods are preferable to identify potential relationships [3, 10, 11]. For instance, Choi et al. [3] introduced a maximum likelihood estimator to estimate the probabilities that a pair of individuals share neither, one or both of their two alleles at a locus being IBD. They used the EM algorithm to find maximum-likelihood estimators. The method-of-moments are more efficient with the growing sample sizes of GWAS studies [12–14]. For example, the sample correlation-based genomic relationship matrix (scGRM) method estimates kinship coefficient by calculating the sample correlation coefficient between a pair of subjects among all single nucleotide polymorphisms (SNPs), in which the observed allele frequencies are used for the calculation of both expectation and variance of genotypes [15, 16]. Some variants of methods based on GRM were also proposed to increase the robustness and efficiency of the estimation, such as the robust GRM (rGRM) [17] and two-step GRM (tsGRM) [18]. KING [19] introduced an alternative and fast moment estimator framework under the random mating assumption and can be extended to population with unknown population structure.

We note that most association mapping and heritability estimation packages use this method as their default setting for calculating GRM, such as GCTA, GEMMA and FaSTLMM [4, 8, 20]. Although this method is widely used, researchers have noted that a substantial proportion of the estimated kinship coefficients are negative, regardless of the actual genetic structure [21]. However, by definition of the kinship coefficient (see “Method” section), negative values from estimators are difficult to interpret, and are treated as due to sampling errors [13, 21–23].

In this paper, through mathematical derivation, we first show that there indeed exists bias in the estimated kinship coefficients using the scGRM method. The bias exists because the observed allele frequencies are regarded as true frequencies. We also prove analytically that the bias essentially results in a negative average for all estimates, which explains the large proportion of negative values. Based on this observation, we propose an improved kinship estimation method, UKin, which can remove bias. We provide a mathematical proof for the unbiasedness of the UKin estimator. Simulations and real data analyses also demonstrate that both bias and standard deviation (SD) can be reduced by replacing the scGRM method with our UKin method. In real data analyses, we apply our method to two studies, young-onset breast cancer (BC) and familial intracranial aneurysm (FIA), which have pedigree information to evaluate our results. For further comparison, we also include another widely used relationship inference method: KING [19], and two other estimators in the framework of GRM: robust GRM (rGRM) [17] and two-step GRM (tsGRM) [18], in our simulations and real data analyses. With an alternative framework, KING provides a robust and efficient relationship estimate. However, as it was pointed out in the original paper, KING becomes less reliable with a small number of SNPs, especially for distant relatives. Results from both simulation and real data analyses suggested that compared with scGRM, rGRM, tsGRM and KING, UKin has lower bias in relationship inference. Besides, UKin performed well for SNP panels from only a few thousand markers to hundreds of thousands of markers.

To further demonstrate the practicability of UKin, we conducted experiments in estimating trait heritability based on real genotype data collected from the young-onset breast cancer (BC) and familial intracranial aneurysm (FIA) studies. Results from these analyses suggest that compared with KING, scGRM, rGRM and tsGRM, UKin achieved more accurate estimation in trait heritability and its performance was also stable with respect to the size of SNP panel.

We summarize the contribution of our method as follows:

1. We prove that the bias exists in the estimation of kinship coefficients using the scGRM method.
2. We analytically show that the bias essentially results in a negative average, explaining the large proportion of negative values in the estimates.
3. We propose an unbiased method for kinship estimation, UKin. We further prove the unbiasedness of the UKin estimator.
4. Simulations and real data applications demonstrate that UKin leads to more accurate estimates compared with the state-of-the-art methods.

The paper is organized as follows. In the “Method” section, we present the theoretical details which show the scGRM is biased, propose our UKin method and give the correctness proof, as well as its connection with the scGRM estimator. In the “Results” section, we evaluate the performance of UKin through several simulations and two real data sets in BC and FIA to validate our theoretical derivation and demonstrate the effectiveness of UKin estimator in reducing bias for relationship inference and heritability estimation. Technical details such as mathematical derivations are provided in Additional file 1.

## Method

Alleles are said to be identical by descent (IBD) if they are inherited from a common ancestor. To describe the average amount of IBD sharing at the genome level, we often adopt the concept of kinship coefficient [18]. For two individuals indexed by *a* and *b*, their kinship coefficient, $\phi _{ab}$, is defined as the probability that two alleles sampled at random from two individuals at the same autosomal locus are IBD. Let $k_{0ab}$, $k_{1ab}$, $k_{2ab}$ denote the probability that individuals *a* and *b* share zero, one and two alleles IBD, respectively. The definition of kinship coefficient indicates that $\phi _{ab}$ can be expressed as a function of those IBD-sharing probabilities, to be more explicit, $\phi _{ab}=k_{1ab}/4+k_{2ab}/2$. Table 1 lists values of kinship coefficients, their corresponding IBD-sharing probabilities and the inference criteria of $\phi _{ab}$ derived using powers of 2 [19] for various relative pairs under the assumption of no inbreeding.

**Table 1:** Kinship coefficients for different relative pairs

Relationship	$\phi _{ab}$	$(k_{0ab},k_{1ab},k_{2ab})$	Inference criteria
MZ twins	0.5	(0, 0, 1)	$>\frac{1}{2^{3/2}}$
Parent-offspring	0.25	(0, 1, 0)	$(\frac{1}{2^{5/2}},\frac{1}{2^{3/2}})$
Full sibs	0.25	(0.25, 0.5, 0.25)	$(\frac{1}{2^{5/2}},\frac{1}{2^{3/2}})$
Half sibs	0.125	(0.5, 0.5, 0)	$(\frac{1}{2^{7/2}},\frac{1}{2^{5/2}})$
Uncle-niece	0.125	(0.5, 0.5, 0)	$(\frac{1}{2^{7/2}},\frac{1}{2^{5/2}})$
First cousin	0.0625	(0.75, 0.25, 0)	$(\frac{1}{2^{9/2}},\frac{1}{2^{7/2}})$
Unrelated	0	(1, 0, 0)	$<\frac{1}{2^{9/2}}$

Suppose we have genotype data of *n* individuals, for each person we consider his/her genotypes at *m* SNP markers respectively. For $1\le i\le n,1\le j\le m$, let $X_{ij}$ be the number of reference alleles (with label *A*) for individual *i* at SNP marker *j*. Thus $X_{ij}$ takes values 0, 1, or 2 according to whether individual *i* has, respectively, 0,1, or 2 copies of allele *A* at marker *j*.

To simplify the illustration, we denote $\mu _j$ and $\sigma ^2_j$ as the expectation and variance of $X_{ij}$, respectively. In other words, $E(X_{ij})=\mu _j, Var(X_{ij})=\sigma ^2_j.$ We assume the population variance for each marker is already known throughout our derivation. In practice, we can use sample variance, an unbiased estimator of population variance, as a substitute. Now we consider a pair of individuals *i* and $i^{'}$. We use $\rho _{ii^{'},j}$ to denote the correlation coefficient between $X_{ij}$ and $X_{i^{'}j}$. Besides, we let $\bar{\rho }_j$ be the average of $\rho _{ii^{'},j}$ among all the individual pairs, i.e.

$$\begin{aligned}\bar{\rho }_j=\frac{\sum \nolimits _{i=1}^{n}\sum \nolimits _{i^{'}=i+1}^{n}\rho _{ii^{'},j}}{n(n-1)/2}.\end{aligned}$$

If we further assume all individuals are sampled from a homogeneous population, we can derive the following relationship among those correlations:

***Property 1.*** Assume all individuals are sampled from a homogeneous population, then for $1\le i, i^{'}\le n, 1\le j\le m$, we have

$$\begin{aligned}&i. \rho _{ii^{'},j}=\rho _{ii^{'}}, \ \bar{\rho }_j=\bar{\rho }.\\&ii. \rho _{ii^{'}}=2\phi _{ii^{'}}. \end{aligned}$$

This property has also been mentioned in other articles, for example, see [16]. A proof of this property is given in Additional file 1. Now we summarize the conclusions of this property as follows:

Result i. implies that the correlation between $X_{ij}$ and $X_{i^{'}j}$ is irrelevant to which SNP we choose and depends only on the pair of individuals we select. Result ii. provides the quantitative relation between the kinship coefficient and the correlation of genotypes, which indicates that the estimation of kinship coefficient $\phi _{ii^{'}}$ is equivalent to estimating the correlation coefficient of genotypes between individual *i* and $i^{'}$ ($\rho _{ii^{'}}$).

Estimating kinship coefficient by calculating the average sample pairwise correlation among all genetic variants has been taken by many methods. Following this principle, a natural estimator of $\rho _{ii^{'}}$ is

1 $$\begin{aligned} \hat{\rho }_{ii^{'}}=\frac{1}{m}\sum \limits _{j=1}^{m}\frac{(X_{ij}-\bar{X}_j)(X_{i^{'}j}-\bar{X}_j)}{\sigma ^2_j}. \end{aligned}$$

where $\bar{X}_j=\frac{1}{n}\sum \nolimits _{i=1}^{n}X_{ij}$ is the average counts of reference alleles (with label *A*) at SNP *j* in the whole population. We call $\hat{\phi }_{ii^{'}}=\frac{1}{2}\hat{\rho }_{ii^{'}}$ the scGRM estimator.

However, as we are going to demonstrate, $\hat{\rho }_{ii^{'}}$ is actually a biased estimator of $\rho _{ii^{'}}$. To illustrate this, we need the following property:

***Property 2.*** For $1\le i, i^{'}\le n,1\le j\le m$, the estimated correlation coefficient between $X_{ij}$ and $X_{i^{'}j}$ has a systematic bias from $\rho _{ii^{'}}$. More specifically, we have

2 $$\begin{aligned}&E\left[ \frac{(X_{ij}-\bar{X}_j)(X_{i^{'}j}-\bar{X}_j)}{\sigma ^2_j}\right]=\rho_{ii^{'}}-\frac{1}{n}\mathop {\sum \limits _{a=1}^{n}}\limits _{a\ne i}\rho _{ia}-\frac{1}{n} \mathop {\sum \limits _{a=1}^{n}}\limits _{a\ne i^{'}}\rho _{ai^{'}}-\frac{1}{n}+\frac{n-1}{n}\bar{\rho }. \end{aligned}$$

The proof is given in Additional file 1.

Equation (2) also reveals that the expected value of $\frac{1}{\sigma ^2_j}(X_{ij}-\bar{X}_j)(X_{i^{'}j}-\bar{X}_j)$ is not related to which SNP we select. Now we consider the expectation of estimator (1), it comes to the conclusion that

$$\begin{aligned} E\hat{\rho }_{ii^{'}}&=E\left[ \frac{1}{m}\sum \limits _{j=1}^{m}\frac{(X_{ij}-\bar{X}_j)(X_{i^{'}j}-\bar{X}_j)}{\sigma ^2_j}\right] \\&=\frac{1}{m}\sum \limits _{j=1}^{m}E\left[ \frac{(X_{ij}-\bar{X}_j)(X_{i^{'}j}-\bar{X}_j)}{\sigma ^2_j}\right] \\&=\rho _{ii^{'}}-\frac{1}{n}\mathop {\sum \limits _{a=1}^{n}}\limits _{a\ne i}\rho _{ia}-\frac{1}{n} \mathop {\sum \limits _{a=1}^{n}}\limits _{a\ne i^{'}} \rho _{ai^{'}}-\frac{1}{n}+\frac{n-1}{n}\bar{\rho }. \end{aligned}$$

If $\hat{\rho }_{ii^{'}}$ is an unbiased estimator of $\rho _{ii^{'}}$, then we should have $E\hat{\rho }_{ii^{'}}=\rho _{ii^{'}}$. However, the result we derive is obviously contradictory to it. The existence of bias means a systematic error when we estimate kinship coefficient via the scGRM method mentioned above. To make this fact clearer, we sum the expectation of $\frac{1}{\sigma ^2_j}(X_{ij}-\bar{X}_j)(X_{i^{'}j}-\bar{X}_j)$ up over all the individual pairs in the population, which leads to the following property:

***Property 3.*** For every SNP marker *j*, where $1\le j\le m$, we have

$$\begin{aligned}E\left[ \sum \limits _{i=1}^{n}\sum \limits _{i^{'}= i+1}^{n}\frac{(X_{ij}-\bar{X}_j)(X_{i^{'}j}-\bar{X}_j)}{\sigma ^2_j}\right] =\frac{n-1}{2}(\bar{\rho }-1).\end{aligned}$$

The proof is given in Additional file 1.

Recall that $E\hat{\rho }_{ii^{'}}=E\frac{1}{\sigma ^2_j}(X_{ij}-\bar{X}_j)(X_{i^{'}j}-\bar{X}_j)$, thus Property 3 also suggests

$$\begin{aligned} \sum \limits _{i=1}^{n}\sum \limits _{i^{'}= i+1}^{n}E\hat{\rho }_{ii^{'}}=\frac{n-1}{2}(\bar{\rho }-1). \end{aligned}$$

From Property 1 we know $\bar{\rho }$ is the theoretical mean value of correlations between pair-wise individuals, therefore it must take the value between 0 and 1. This fact together with Property 3 reveals that the mean value of estimator $\hat{\rho }_{ii^{'}}$ is negative on average, which explains the empirical observation that a substantial proportion of estimated kinship coefficients are negative.

Several GRM estimators have been proposed based on the scGRM method. The robust GRM (rGRM) estimator replaces the equal weights in scGRM with varied weights proportional to $\sigma _{j}^{2}$, while two-step GRM (tsGRM) improves the scGRM estimator by selecting the one with minimum variance from a general class of GRM estimators [18]. Both rGRM estimator and tsGRM estimator can also be proved to be biased, with details given in Additional file 1.

This bias problem makes the scGRM estimator $\hat{\phi }_{ii^{'}}$ less desirable as an estimator of kinship between individuals *i* and $i^{'}$. We can design an improved kinship estimation method which can eliminate the bias for each pair of individuals based on the scGRM estimator $\hat{\phi }_{ii^{'}}$. The improved estimation method, UKin, which stands for the unbiased kinship estimator, solves the bias problem without adding much computational complexity. To understand how this method guarantees the unbiasedness, we need the following property:

***Property 4.*** For every SNP marker $j, 1\le j\le m$, and every pair of individuals *i* and $i^{'}$, $1\le i$, $i^{'}\le n$, we have

3 $$\begin{aligned}&E\left[ \frac{(X_{ij}-\bar{X}_j)(X_{i^{'}j}-\bar{X}_j)}{\sigma ^2_j} +\frac{1}{2}\mathop {\sum \limits _{k=1}^{n}}\limits _{k\ne i}\frac{(X_{ij}-\bar{X}_j)(X_{kj}-\bar{X}_j)}{\sigma ^2_j}+\frac{1}{2}\mathop {\sum \limits _{l=1}^{n}}\limits _{l\ne i^{'}}\frac{(X_{lj}-\bar{X}_j)(X_{i^{'}j}-\bar{X}_j)}{\sigma ^2_j}+1\right] =\rho _{ii^{'}}. \end{aligned}$$

The proof is given in Additional file 1.

For ease of presentation, we set

$$\begin{aligned} u_{ii^{'}}^j&=1+\frac{1}{2}\mathop {\sum \limits _{k=1}^{n}}\limits _{k\ne i}\frac{(X_{ij}-\bar{X}_j)(X_{kj}-\bar{X}_j)}{\sigma ^2_j}+\frac{1}{2}\mathop {\sum \limits _{l=1}^{n}}\limits _{l\ne i^{'}}\frac{(X_{lj}-\bar{X}_j)(X_{i^{'}j}-\bar{X}_j)}{\sigma ^2_j}+\frac{(X_{ij}-\bar{X}_j)(X_{i^{'}j}-\bar{X}_j)}{\sigma ^2_j}. \end{aligned}$$

Using (3), we also conclude that the expectation of $u_{ii^{'}}^j$ does not depend on which SNP we select. Based on this fact, a reasonable estimator of $\rho _{ii^{'}}$ is

4 $$\begin{aligned} \tilde{\rho }_{ii^{'}}=\frac{1}{m}\sum \limits _{j=1}^{m}u_{ii^{'}}^j. \end{aligned}$$

As Property 4 shows $Eu_{ii^{'}}^j=\rho _{ii^{'}}$ holds for every $1\le j\le m$, the expectation of $\tilde{\rho }_{ii^{'}}$ is still $\rho _{ii^{'}}$. In other words, $\tilde{\rho }_{ii^{'}}$ is an unbiased estimator of $\rho _{ii^{'}}$, thus $\tilde{\phi }_{ii^{'}}=\frac{1}{2}\tilde{\rho }_{ii^{'}}$ is an unbiased kinship estimator. Besides, as we can observe from the expression of $Eu_{ii^{'}}^j$ , $\tilde{\rho }_{ii^{'}}$ is the sum of a group of scGRM estimators $\hat{\rho }_{ii^{'}}$ and a few correction terms, which means the UKin estimator relies on the same information we need for calculating the scGRM estimator $\hat{\phi }_{ii^{'}}$. Thus the implementation of the UKin method doesn’t require extra data.

It is worth noting that there exists some relationship between the scGRM and UKin estimator. Substituting the expression of $u_{ii^{'}}^j$ into (4), we get

5 $$\begin{aligned} \tilde{\rho }_{ii^{'}}=\hat{\rho }_{ii^{'}}+\frac{1}{2} \mathop {\sum \limits _{k=1}^{n}}\limits _{k\ne i}\hat{\rho }_{ik}+\frac{1}{2} \mathop {\sum \limits _{l=1}^{n}}\limits _{l\ne i^{'}} \hat{\rho }_{li^{'}}+1. \end{aligned}$$

Equation (5) indicates that the UKin estimator $\tilde{\phi }_{ii^{'}}$ is a linear combination of some scGRM estimators $\hat{\phi }_{ii^{'}}$ and constants. Thus $\tilde{\phi }_{ii^{'}}$ and $\hat{\phi }_{ii^{'}}$ are based on the same genetic information. Besides, this conclusion also shows that the UKin method won’t bring a significant increase in computational complexity than the scGRM method.

Throughout our above analysis, we make assumptions of no inbreeding, LE and population homogeneity. In the Discussion we analyzed these assumptions in detail.

## Results

### UKin reduces bias in simulation studies

#### An illustrative example

We start our discussion with a simple but extreme example. In this experiment, we assumed that there were 500 full siblings from the same family. Although unlikely to exist in reality, this example serves as a good illustration of our theoretical derivation. As every two individuals selected from the same family were full siblings, the true value of their kinship coefficient should be 0.25 (see in Table 1). However, following Property 3 in the “Method” section, their average kinship coefficient estimated by scGRM, denoted by $\bar{\hat{\phi }}$, should have the expectation:

$$\begin{aligned}E\bar{\hat{\phi }}=\frac{n-1}{4}(\bar{\rho }-1)/\left( \frac{n(n-1)}{2}\right) =\frac{\bar{\rho }-1}{2n}=\frac{0.5-1}{2\times 500}=-5\times 10^{-4},\end{aligned}$$

where *n* is the sample size and $\bar{\rho }$ is the average of their true genetic correlation coefficients. Property 1 together with Table 1 in the “Method” section suggest that $\bar{\rho }=0.5$ for full siblings.

**Fig. 1:**
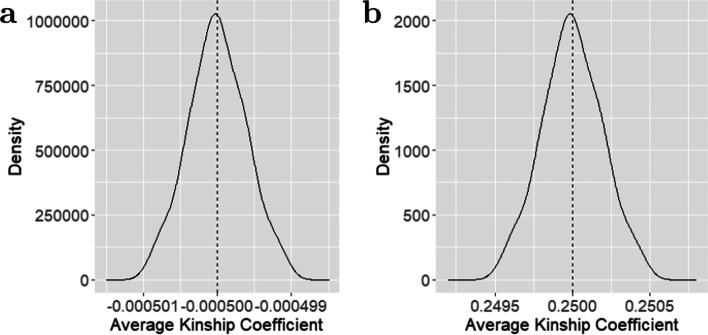
Distribution of average kinship coefficients estimated by the scGRM (**a**) and UKin (**b**). Two hundred unrelated families each consisting of 500 full siblings were simulated, with each sibling genotyped at 10,000 SNPs. The averages of kinship coefficients among all individual pairs from the same family were calculated and the distribution of these averages is displayed. The true value of kinship coefficient between full siblings is 0.25. The vertical dashed line in each plot corresponds to the mean value of these averages estimated by the corresponding method

This result shows the unexpected phenomenon that although all individuals in our simulated samples are full siblings to each other, the average of the estimated kinship coefficients has a negative value. To illustrate Property 3 in practice, we simulated 200 unrelated families each consisting of 500 full siblings with the method provided by the package CorBin [24]. Each individual was genotyped at 10,000 SNPs. Following the scGRM method and the UKin method proposed in the “Method” section, we estimated pairwise kinship coefficients and calculated their mean values, respectively. The histograms of these estimated average kinship coefficients are shown in Fig. 1. From this plot, we could see the distribution of average kinship estimated by the scGRM method centered around $-5\times 10^{-4}$, which is consistent with our expectation from the analytical results. By contrast, the UKin approach performed better in dealing with this extreme situation, with the average estimates centered at 0.25, the true value of pairwise kinship coefficient for full-sibling pairs. Besides, from Fig. 1 we could observe that the two distributions have similar shapes, which could be explained by Eq. (5) in the “Method” section which suggests that unbiased estimator of correlation coefficient $\tilde{\rho }_{ii^{'}}$ could be expressed as a linear combination of the scGRM estimators $\hat{\rho }_{ii^{'}}$. Considering there were 500 full siblings from the same family, we calculated the average on both sides of Eq. (5) among all the simulated individual pairs, which is $\bar{\tilde{\rho }}=500\bar{\hat{\rho }}+1$, where $\bar{\tilde{\rho }}$ and $\bar{\hat{\rho }}$ represent the average of correlation coefficients between full siblings from the same family, estimated by the UKin method and the scGRM method respectively, i.e.

$$\begin{aligned} \bar{\tilde{\rho }}=\frac{\sum \nolimits _{i=1}^{n}\sum \nolimits _{i^{'}=i+1}^{n}\tilde{\rho }_{ii^{'}}}{n(n-1)/2} \qquad \qquad \bar{\hat{\rho }}=\frac{\sum \nolimits _{i=1}^{n}\sum \nolimits _{i^{'}=i+1}^{n}\hat{\rho }_{ii^{'}}}{n(n-1)/2}. \end{aligned}$$

As there was a linear relationship between kinship coefficient and correlation coefficient (see Property 1 in the “Method” section), the distributions of the average kinship coefficients estimated by the two methods should have the same shape.

#### A more general simulation

To evaluate the performance of UKin in kinship coefficient estimation in a more general situation, we performed the following simulations in which population homogeneity was assumed. To include different relationships in our experiment, we simulated 4000 people including 500 pairs with kinship coefficient 0.125, 500 pairs with coefficient 0.25, and 500 pairs with coefficient 0.5. For simplicity, different relative pairs were set to be unrelated. In addition, we also included 1000 people who had no relationship with other individuals. For each subject, genotype data were generated for 50,000 random and independent SNPs. The minor allele frequencies (MAFs) of genotyped variants were drawn uniformly from [0.05, 0.5].

**Fig. 2:**
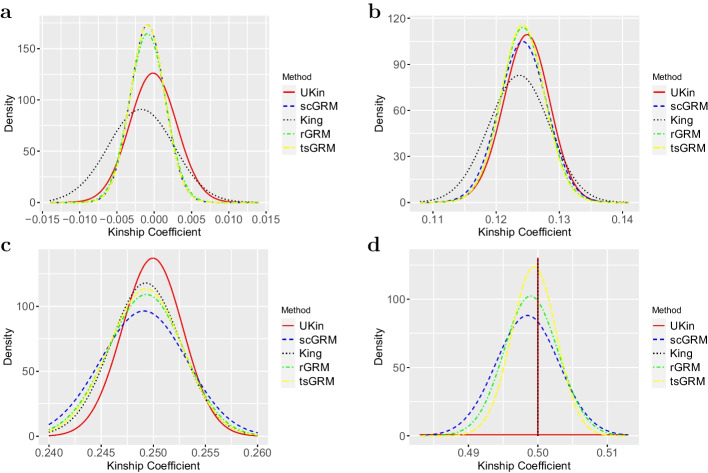
Distributions of kinship coefficients estimated by UKin, scGRM, KING, rGRM and tsGRM. This simulation study includes 4000 individuals with different relationships. The four plots correspond to the four groups divided by the true value of estimated kinship coefficients: 0 (**a**), 0.125 (**b**), 0.25 (**c**), 0.5 (**d**). Genotype data of 50,000 random SNPs are generated for each subject

We compare UKin with scGRM, KING, and two other GRM estimators: rGRM and tsGRM. With each estimator, we estimated kinship coefficients between all simulated individual pairs and divided those coefficients into four groups according to their true relationships. Figure 2 shows the distribution of the estimated kinship coefficients in each group respectively. As shown in this plot and summarized in Table 2, for groups with true kinship coefficients 0.25 and 0.5, UKin achieved the lowest standard deviation (SD) of estimated kinship among the three relationship inference methods. For true kinship coefficient 0.125 and independent pairs, tsGRM performed the best in reducing the SD. It is also worth mentioning that KING has the largest bias and SD for independent pairs, which might has a negative impact on the application of KING.

Although Fig. 2 clearly reflects the SDs for five methods, it is difficult to compare their biases from the plots. More detailed comparisons are shown in Table 2, where the numbers in bold represent the smallest bias or standard deviation achieved among all the methods. As shown in the left part of this table, UKin always performed better than other methods when we compared the mean values of estimated kinship coefficients, as the estimates of the UKin method were much closer to true values for all four groups. Besides, Table 2 also indicates that UKin and KING show a downward trend of biases and SDs with increasing true kinship coefficients, which suggests that UKin and KING tend to get more accurate inference for close relatives. For general relationship, UKin was always superior to KING in reducing both estimation bias and SD. In contrast, all three GRM estimators achieved smaller SDs for unrelated individual pairs but performed poorly regarding to close relatives. It is also notable that when we considered individual pairs with kinship coefficient 0.5, i.e., monozygotic twins (MZ twins), both bias and SD were extremely close to zero if we utilize UKin or KING to estimate.

**Table 2:** Comparison of UKin, KING, scGRM, rbGRM, and tsGRM in biases and SDs (50,000 SNPs)

True value	Bias from True Value $(\times 10^{-3})$					Standard deviation $(\times 10^{-3})$				
	UKin	KING	scGRM	rbGRM	tsGRM	UKin	KING	scGRM	rbGRM	tsGRM
0.000	− **0.152**	− 1.810	− 0.898	− 0.899	− 0.899	3.101	4.317	2.262	2.381	**2.261**
0.125	− **0.227**	− 1.405	− 0.730	− 0.715	− 0.781	2.595	3.346	2.654	2.530	**2.449**
0.250	− **0.081**	− 0.703	− 0.953	− 0.786	− 0.793	**2.020**	2.428	2.864	2.557	2.432
0.500	**0.000**	**0.000**	− 1.492	− 0.961	− 0.609	**0.000**	**0.000**	3.171	2.718	2.254

We also conducted a small-panel simulation including 4000 subjects and 10,000 SNPs to evaluate the performance of UKin, scGRM and KING when the number of genotyped SNPs is relatively small. The population structure was the same as the previous simulation. Summarized in Table A1 in Additional file 1, KING had the worst performance among three methods in all relationships except MZ twins because of its largest bias and SD. This result shows that compared to UKin and scGRM, KING was poor at handling small SNP panels. As also pointed out in [19], KING requires a large SNP panel to make accurate estimation. A panel with thousands of SNPs could cause a decrease in overall accuracy and only allowed KING to identify closely related pairs. This drawback makes KING less efficient in dealing with small dataset. In comparison, UKin achieved a much more stable performance even when the number of genotyped SNPs is small.

### UKin reduces bias in real data applications

#### The young-onset breast cancer study

To demonstrate UKin could get more accurate results in estimating kinship coefficients in real applications, we applied it to a family-based study of genes and environment in young-onset BC (*dbGaP Study Accession: phs000678.v1.p1*). This study recruited families from the US and Puerto Rico with a daughter who was recently diagnosed with breast cancer and another unaffected daughter. For each family, only the diseased daughter and her unaffected full sister were genotyped for analysis. As for data quality control, we removed individuals with more than 10% missing genotypes as well as SNPs with a missing genotype rate greater than 5% or a minor allele frequency less than 5%. After further removing individuals with missing phenotypes, we got 1983 subjects (1458 cases and 525 controls) with 925,685 variants in total. The processed data included 500 pairs of full sisters, with one affected by breast cancer. Based on Table 1, the true values of estimated kinship coefficients should be 0.25 for these full sister pairs.

We first applied the scGRM method to estimate the kinship coefficients, which had poor performance. As shown in Table 3, where the estimated numbers of the 1st-degree relative pairs by different methods are in bold, for the 500 pairs of full siblings, only 472 pairs were estimated to have kinship coefficients between $2^{-5/2}$ and $2^{-3/2}$, which means 5.6% of full sisters were incorrectly inferred to be other kinds of relative pairs. The rGRM method had the same result with scGRM. The tsGRM estimator performed better than scGRM and rGRM with 17 1st-degree relative pairs misspecified as MZ twins. In contrast, both UKin and KING identified all the full sisters pairs correctly. Besides, scGRM misspecified 4721 unrelated pairs as full siblings, suggesting estimates of scGRM showed a obvious distribution overlap between full siblings and unrelated pairs. However, both UKin and KING did not make such mistakes, which indicates that UKin and KING performed better in separating relatives from unrelated pairs.

**Table 3:** Distribution of estimated kinship coefficients of 500 full siblings in the BC study

Relationship	Unrelated relative pairs	3rd-degree relative pairs	2nd-degree	**1st-degree relative pairs**	MZ twins
Inference criteria	$<\frac{1}{2^{9/2}}$	$(\frac{1}{2^{9/2}},\frac{1}{2^{7/2}})$	$(\frac{1}{2^{7/2}},\frac{1}{2^{5/2}})$	$\mathbf {(\frac{1}{2^{5/2}},\frac{1}{2^{3/2}})}$	$>\frac{1}{2^{3/2}}$
UKin	0	0	0	**500**	0
KING	0	0	0	**500**	0
scGRM	0	0	0	**472**	28
rGRM	0	0	0	**472**	28
tsGRM	0	0	0	**483**	17
True	0	0	0	**500**	0

**Fig. 3:**
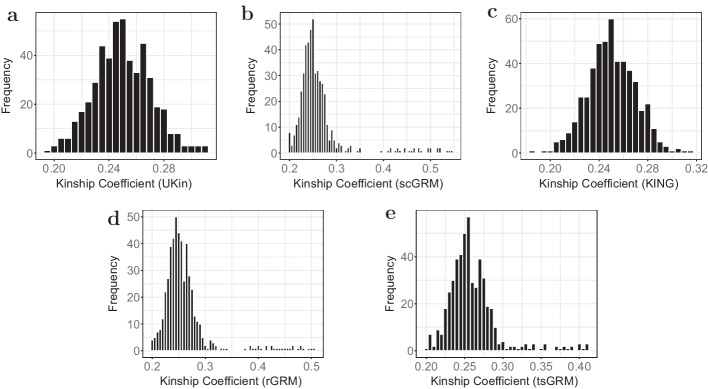
Distributions of kinship coefficients estimated by UKin (**a**), scGRM (**b**), KING (**c**), rGRM (**d**) and tsGRM (**e**) in BC study. This study genotyped 1983 individuals at 925,685 variants. In this figure, we only considered estimated kinship coefficients of 500 full sister pairs from irrelevant families. Class interval of the histogram for each method is set to be 0.005

**Fig. 4:**
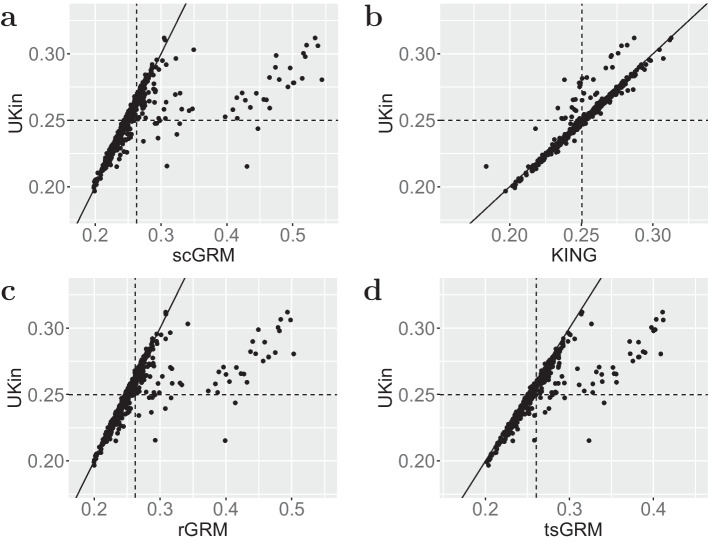
Scatter plot of estimated kinship coefficients in BC study. For this plot we only consider 500 full sister pairs in the BC data set. We display the scatter plots between UKin and scGRM (**a**), UKin and KING (**b**), UKin and rGRM (**c**), UKin and tsGRM (**d**). The oblique solid line stands for the equation $y=x$, while the vertical and horizontal dashed lines represent the mean values of estimates for the corresponding method, respectively

The histograms of kinship coefficients estimated by UKin, KING, and three GRM estimators for the 500 full sister paris in the BC study are given in Fig. 3. It is obvious that the histograms corresponding to GRM methods contain more pairs with estimated kinship coefficients larger than $2^{-3/2}$, which means these full siblings are misspecified as MZ twins. In contrast, UKin and KING are much less likely to make such mistakes. In Table 4 we display the bias and SD of 500 estimated kinship coefficients for full sisters, with the smallest bias and SD in bold. Obviously, UKin performed best in reducing estimation bias, but had a slightly larger SD than KING. The GRM methods, by contrast, performed poorly on both counts. To visualize the difference among these methods, we also draw the scatter plots of the estimated kinship coefficients for the 500 full sister pairs between UKin and scGRM (Fig. 4a), UKin and KING (Fig. 4b), UKin and rGRM (Fig. 4c), UKin and tsGRM (Fig. 4d). The scatter plots demonstrated that while distributions of UKin and KING estimates were similar and closed to true value, three GRM methods overestimated the kinship coefficients for many full sister pairs.

**Table 4:** Bias and SD of estimated kinship coefficients in BC study

Estimation method	UKin	KING	scGRM	rGRM	tsGRM
Bias$(\times 10^{-3})$	− **0.053**	0.355	12.815	12.271	10.400
SD$(\times 10^{-2})$	2.064	**1.979**	5.685	4.913	3.363

#### The familial intracranial aneurysm linkage study

To further investigate the effectiveness of the UKin method in kinship coefficient estimation, we applied UKin to infer pedigree structure using genotype data from the FIA linkage study (*dbGaP Study Accession: phs000293.v1.p1*). This study recruited 400 families with multiple individuals who have an intracranial aneurysm (IA) through 23 (25) referral centers throughout North America, Australia, and New Zealand that represent 35 (40) recruitment sites. After standard quality control and discarding subjects with missing phenotypes, we obtained 990 individuals from 371 families and each of them was genotyped at 5505 SNPs. In this FIA dataset, the confirmed relationships include 137 first-degree relative pairs (including 19 full siblings and 118 parent-child pairs).

We compared the performance of UKin, KING, and three GRM methods in identifying these first-degree relative pairs and estimating their kinship coefficients. As shown in Table 5, where the estimated numbers of the 1st-degree relative pairs by different methods are in bold, UKin and KING were able to correctly recognize all the 137 first-degree pairs (with estimated kinship coefficients between $2^{-2.5}$ and $2^{-1.5}$), while scGRM misspecified one parent-child pair as MZ twins, with an estimated kinship coefficient of 0.442. This parent-child pair was also incorrectly classified to MZ twins by rGRM and tsGRM.

**Table 5:** Distribution of estimated kinship coefficients of 137 first-degree relative pairs in FIA study

Relationship	**Unrelated relative pairs**	3rd-degree relative pairs	2nd-degree relative pairs	1st-degree relative pairs	MZ twins
Inference criteria	$\mathbf {<\frac{1}{2^{9/2}}}$	$(\frac{1}{2^{9/2}},\frac{1}{2^{7/2}})$	$(\frac{1}{2^{7/2}},\frac{1}{2^{5/2}})$	$(\frac{1}{2^{5/2}},\frac{1}{2^{3/2}})$	$>\frac{1}{2^{3/2}}$
scGRM	0	0	0	**136**	1
UKin	0	0	0	**137**	0
KING	0	0	0	**137**	0
rGRM	0	0	0	**136**	1
tsGRM	0	0	0	**136**	1
True	0	0	0	**137**	0

**Fig. 5:**
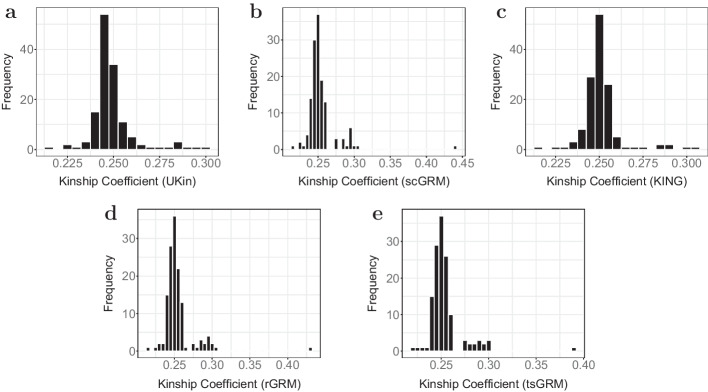
Distributions of estimated kinship coefficients in the FIA study with UKin (**a**), scGRM (**b**), KING (**c**), rGRM (**d**) and tsGRM (**e**). Among all the 137 first-degree relative pairs in this dataset, there are 19 full siblings and 118 parent-child pairs. Class interval of the histogram for each method is set to be 0.005

**Fig. 6:**
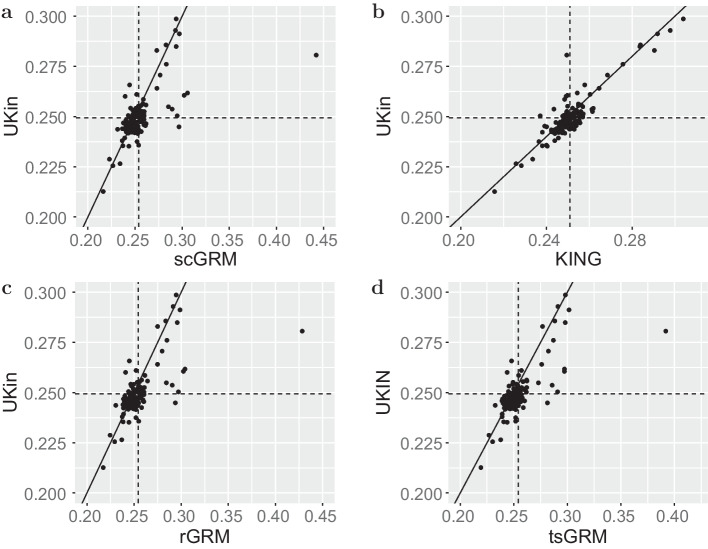
Scatter plot of the estimated kinship coefficients in FIA study. Only the 137 first-degree pairs are shown. We display the scatter plots between UKin and scGRM (**a**), UKin and KING (**b**), UKin and rGRM (**c**), UKin and tsGRM (**d**). The oblique solid line stands for the equation $y=x$, while the horizontal and vertical dashed lines correspond to the mean values of estimates by UKin and comparing method, respectively

The histograms of the kinship coefficients of these 137 individual pairs estimated by five methods (Fig. 5) indicate that unbiased estimations were more concentrated, taking values between 0.21 and 0.3. However, the distribution of GRM estimations was more dispersed with a distinct outlier. This fact is also shown in the scatter plots including all the 137 first-degree pairs in the FIA data set (Fig. 6). We further calculated the bias from the true value (0.25) and SD of the estimated coefficients for each estimator. As summarized in Table 6, the UKin estimator achieved the least absolute bias among the five methods. The estimation bias of UKin was 1/6 of the bias estimated by scGRM, while the SD of UKin was half of scGRM. We also noted that scGRM misspecified 15 parent-child pairs or unrelated pairs as MZ twins, while UKin only made five such mistakes which were all included in the misspecified pairs of scGRM. Results from the BC and FIA studies indicated that UKin achieved accurate results in relationship inference for both dense SNP panel and small dataset.

**Table 6:** Bias and SD of estimated kinship coefficients in the FIA study

Estimation method	UKin	KING	scGRM	rGRM	tsGRM
Bias $(\times 10^{-3})$	− **0.667**	− 1.039	4.045	4.461	4.224
SD $(\times 10^{-2})$	1.178	**1.139**	2.216	2.126	1.859

The bold numbers stand for the smallest bias or standard deviation among all the methods

In simulations, we showed that UKin always outperformed scGRM and KING in reducing bias of the kinship coefficients estimates. In real data applications, scGRM and other scGRM-based methods failed in identifying all full siblings. As estimates falling in $2^{-5/2}$ and $2^{-3/2}$ were all treated as correct identifications of full siblings, we did not observe differences between UKin and KING in terms of correct identifications. However, UKin still showed smallest biases among all methods in the two real data applications. Besides, a biased estimate of kinship coefficients will further lead to biased estimates in downstream analysis, such as the estimates of heritability. In the following section, we will demonstrate this point using the BC and FIA datasets.

### Experiments on heritability estimation

Heritability is an important parameter that measures the proportion of phenotypic variance that is attributable to additive genetic factors. Estimation of heritability is optimally achieved in pedigree-based GWAS, where inference of genomic relatedness plays a key role [25]. In order to evaluate the performance of UKin in heritability estimation and compare it with scGRM, rGRM, tsGRM and KING, we conducted simulations based on the BC and FIA studies and used genomic-relatedness-matrix restricted maximum likelihood (GREML) implemented in the GCTA software tool [4] to analyze.

#### The young-onset breast cancer study

In the first experiment, we used the genotype data in the young-onset BC study and simulated phenotypes with pre-set heritability. After standard quality control, 1983 individuals and 925,685 SNPs remained for analysis. We set the true heritability to be one of the five values: 0.3, 0.4, 0.5, 0.6, 0.7 and the proportion of risk SNPs proportion to be 0.01. With the kinship matrix generated by three methods (UKin, KING and scGRM), we use GCTA-reml to estimate the heritability and its standard error via one of the three kinship estimation methods. For each true heritability value, we repeated this experiment for 1000 times.

**Fig. 7:**
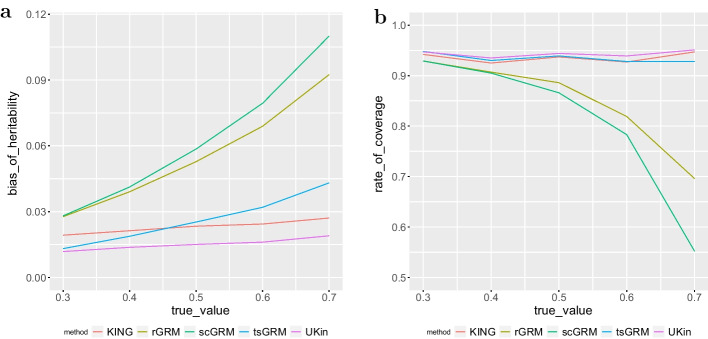
Bias of estimated heritability (**a**) and coverage rate of 95% confidence interval (**b**) in the BC study. For each true heritability setting (0.3, 0.4, 0.5, 0.6, 0.7), we repeated the simulation for 1000 times. For each time we used the estimated heritability and SD to construct 95% normal CI and calculated the total coverage rate with five relationship estimation methods

We calcualted the average bias and SD of estimated heritability for each true value, with the results summarized in Table 7. The numbers in bold represent the smallest biases achieved among all the methods for different true heritabilities. SDs of estimated heritability were close for five methods, with all these SDs between 0.055 and 0.079. In comparision, these methods performed differently regarding to estimation bias. As shown more clearly in Fig. 7a, we observed the following relative performance of the three methods with respect to the average bias of heritability:

$$\begin{aligned} bias_{UKin}< bias_{KING}< bias_{rGRM} < bias_{scGRM} \end{aligned}$$

The bias of tsGRM method was smaller than KING with a true heritability of 0.5, but was always larger than the bias of UKin. From this plot we also observed that three GRM methods failed to control the bias when the true heritability went up. To be more specific, the estimation bias of scGRM method was always above 0.028, and was larger than 0.11 when the true heritability was 0.7. The tsGRM method suffered less from this problem, but it also showed an evident upward trend with the increasing of true heritability. Compared with GRM methods, biases for UKin and KING were much more stable. When the true heritability changed from 0.3 to 0.7, the bias of UKin increased from 0.012 to 0.019, while the bias of KING increased from 0.019 to 0.027. In other words, the bias of KING was always about 0.08 larger than the bias of UKin.

**Table 7:** Biases and SDs of estimated heritability for five methods on the BC dataset

True value	0.3	0.4	0.5	0.6	0.7
UKin	**0.0118 (0.0729)**	**0.0137 (0.0782)**	**0.0151 (0.0743)**	**0.0161 (0.0715)**	**0.0190 (0.0665)**
KING	0.0193 (0.0722)	0.0213 (0.0784)	0.0234 (0.0742)	0.0244 (0.0716)	0.0271 (0.0668)
scGRM	0.0281 (0.0713)	0.0412 (0.0721)	0.0586 (0.0675)	0.0794 (0.0633)	0.1100 (0.0555)
rGRM	0.0277 (0.0712)	0.0391 (0.0730)	0.0528 (0.0689)	0.0689 (0.0654)	0.0925 (0.0581)
tsGRM	0.0131 (0.0726)	0.0188 (0.0762)	0.0253 (0.0721)	0.0320 (0.0690)	0.0431 (0.0628)

For scGRM and rGRM, large bias and small SD of estimated heritability were likely to result in low coverage of the confidence interval (CI). To demonstrate this, we constructed 95% normal CIs with estimated heritability and corresponding standard error for each simulation and calculated the proportion that CIs intervals covered the true value. We display the coverage rate for each group and method in Fig. 7b. When considering 95% normal CI, UKin, KING, and tsGRM showed good performances, but the coverage rate of UKin interval was always better than results of KING and tsGRM, and was most close to 95%. We also note that scGRM and rGRM performed poorly, especially when the true heritability was large.

#### The familial intracranial aneurysm linkage study

To further compare the effectiveness of UKin in heritability estimation with the other four methods when the number of SNPs is limited, we also conducted similar experiments based on the familial IA linkage study. For the FIA dataset, we have 5505 SNPs and 990 individuals after the QC procedure. We also set the true heritability to be 0.3, 0.4, 0.5, 0.6, or 0.7 and estimated the heritability under UKin, KING, and three GRM methods, respectively.

**Fig. 8:**
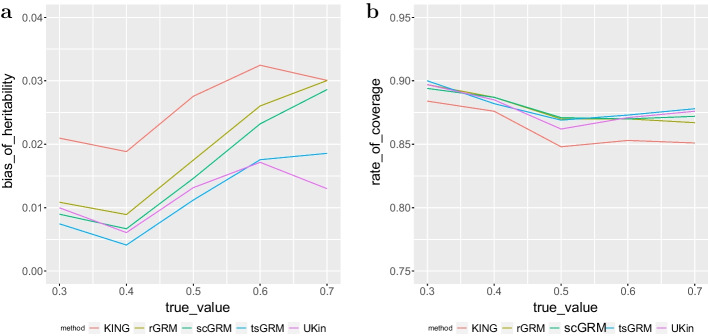
Bias of estimated heritability (**a**) and coverage rate of 95% confidence interval (**b**) in the FIA study. For each true heritability setting (0.3, 0.4, 0.5, 0.6, 0.7), we repeated the simulation for 1000 times. For each time we used the estimated heritability and SD to construct 95% normal CI and calculated the total coverage rate with five relationship inference methods

Biases of heritability estimates and SDs from 1000 simulations are shown in Table 8. The numbers in bold represent the smallest biases achieved among all the methods for different true heritabilities. Compared with the BC simulation, the performance of KING was much worse as the estimation bias was always the largest among all methods. This result further suggested that KING required a large number of genetic markers for accurate inference of relatedness. By contrast, UKin showed a more stable performance with a small number of SNPs. When the true heritability was relatively modest (0.3, 0.4, 0.5), GRM methods and UKin had similar results. However, as shown more clearly in Fig. 8a, both rGRM and scGRM failed to control the estimation bias for high heritabilities. Besides, our results also suggest that the SDs of five methods were similar, with values between 0.067 and 0.081.

**Table 8:** Biases and SDs of estimated heritability for five methods in FIA dataset

True value	0.3	0.4	0.5	0.6	0.7
UKin	0.0099 (0.0747)	0.0061 (0.0791)	0.0132 (0.0809)	**0.0171 (0.0756)**	**0.0130 (0.0725)**
KING	0.0210 (0.0735)	0.0188 (0.0779)	0.0276 (0.0799)	0.0325 (0.0746)	0.0301 (0.0723)
scGRM	0.0090 (0.0742)	0.0067 (0.0776)	0.0146 (0.0787)	0.0232 (0.0726)	0.0286 (0.0670)
rGRM	0.0109 (0.0741)	0.0089 (0.0775)	0.0175 (0.0790)	0.0260 (0.0728)	0.0300 (0.0682)
tsGRM	**0.0074 (0.0746)**	**0.0041 (0.0783)**	**0.0112 (0.0796)**	0.0176 (0.0738)	0.0186 (0.0693)

Similar to the above experiment based on BC, we also constructed 95% normal CIs with estimated heritability and standard error for each simulation and displayed the coverage rate for each method in Fig. 8b. This plot illustrates that both UKin and three GRM methods achieved a stable coverage, and there was little difference between these four methods. By contrast, KING performed poorly in convering the true heritability because of the large estimation bias. However, it was worth noting that none of these approaches achieved the desired 95% coverage, suggesting that limited number of SNPs made all these methods less efficient and KING was influenced the most.

## Discussion

Among many kinship estimation methods, the most commonly applied estimator uses dense SNP genotypes and allele frequencies in samples to calculate average pairwise correlation coefficients among SNPs. Although this method is intuitive and easy to calculate, we have shown in this manuscript that it is actually biased because it treats the observed allele frequencies as true frequencies. Through rigorous derivation, we showed that pairwise kinship coefficients estimated by scGRM add up to be a negative value, which explains the phenomenon that a substantial proportion of kinship coefficient estimates are negative.

When conducting large scale estimates of kinship coefficients, the existing bias in scGRM can lead to incorrect inference of relationships, and this problem can be more severe if the subjects in the dataset are closely related. Our method, UKin, solved this issue by incorporating genetic information from the whole population to adjust for the bias in estimated kinship coefficient between every single pair. This unbiased estimator can be expressed as a polynomial of scGRM estimators, and leveraging only information of dense genotypes from the population. As demonstrated by our simulations and applications to the BC and FIA family data, UKin performed better in reducing both estimation biases and SDs. For the two sister study, the results suggest that while scGRM could lead to severe spurious inference of relative pairs, UKin rarely made such mistakes. Even when the number of genotyped SNPs was limited for the FIA study, UKin could reduce statistical bias and SD while avoiding spurious relationship inference.

### Limitations of the current study

In our theoretical derivations and simulation studies, we made assumptions like linkage equilibrium (LE) and absence of inbreeding, that is, genotypes at different markers are independent. During our derivation, we used the same weights for all SNPs, and our simulated datasets were also generated under this assumption. Although there is linkage disequilibrium (LD) in real data, empirical results from analyses of the BC and FIA family data suggest that UKin could reduce bias in the presence of linkage disequilibrium. To incorporate LD in practice, we can give different weights based on LD among different SNPs. Following the approach of Wang [21], these LD weights ${\textbf {w}}=(w_{1},w_{2},\ldots ,w_{m})^{T}$ can be calculated by solving the following minimization problem:

$$\begin{aligned} \mathop {min}\limits _{{\textbf {w}}}[{\textbf {w}}^{T}{} {\textbf {R}}{} {\textbf {w}}-{\textbf {w}}^{T}1]\ \ : \ \ w_{l}\ge 0, \forall l, \end{aligned}$$

where ${\textbf {R}}=[\rho ^{2}_{lk}]$ is a matrix consisting of squared LD correlations. Theoretically, this result can be directly applied to UKin by assigning the correlation coefficient at each SNP marker its corresponding weight, which might make our approach adapt to LD situation.

Another assumption throughout our study is a homogeneous population so that the allele frequencies can be calculated once and applied to all subjects. Some methods have been proposed to estimate kinship coefficients in admixed populations, where the assumption of population homogeneity is untenable [16, 19, 26]. However, as most of these methods are based on the scGRM method, they are also likely to be biased estimators, too. How to extend our UKin method to deal with admixed populations is a topic for future studies.

Similar to scGRM and rGRM, UKin also has a quadratic time complexity. When dealing with real data, our python script of UKin took 6 seconds to estimate the kinship matrix for the FIA study, while it took 46.4 minutes to finish the calculation with a single CPU core for the large BC study. To improve the computational speed of UKin, we applied parallel execution to our original python code. When multiprocessing UKin with 12 CPU cores, the required computational time for kinship estimation of BC study was reduced to 11.2 minutes. It is also worth mentioning that although the tsGRM estimator shows the best performance in kinship and heritability estimation among all three GRM methods, it requires much longer running time because tsGRM needs to make optimization based on a pre-calculated kinship matrix. When calculating the kinship matrix for the BC dataset with a single CPU core, tsGRM spent about 46 hours to complete, which is far from satisfying. Implementing our UKin method using GPU will further improve the computational efficiency of the method. In this paper, we mainly focus on the concept of correcting bias of kinship estimation method. We will consider accelerating the algorithm in our future work.

### Future works

Beside to overcome the current limitations of our method mentioned above, further studies need to be conducted for demonstrating how more accurate kinship estimation will benefit downstream analyses based on genetic data.

Results show that UKin achieved more stable and accurate estimation of heritability compared with other GRM methods and KING. In addition, more accurate kinship estimation will improve the performance of other genetic analyses such as association mapping. In recent years, GWAS have seen great success in identifying genetic loci contributing to complex human traits [27, 28]. By studying a genome-wide data set of genetic variants in different individuals, GWAS looks for SNPs correlated with traits in the samples. Accurate specification of familial relationships is expected to bring more powerful association results in GWAS with unknown family structure.

To demonstrate whether the change of kinship matrix affects the performance of association mapping, we conducted a simulation study to compare the performance of UKin, scGRM, and KING in GWAS. In our experiments, we simulated 4000 samples including 2000 cases and 2000 controls. We included subjects with various pairwise kinship coefficients in both cases and controls. More specifically, we simulated 250 first-degree relative pairs, 250 2nd-degree relative pairs, 250 3rd-degree relative pairs, and 500 unrelated subjects for both cases and controls. The total number of SNPs genotyped for each individual was set to be 10,000 and the MAFs of non-risk SNPs were drawn uniformly from $[0.05,\ 0.5]$. The proportion of risk SNPs was set at 0.05. For these risk SNPs, a variable following the Gaussian distribution $\mathcal {N}(0,\ 0.05^{2})$ was added to the previous uniform distribution to obtain their MAFs in cases. We set those MAFs below 0.05 or greater than 0.95 to be 0.05 and 0.95, respectively.

We applied GEMMA [20], which was developed to implement the genome-wide mixed model association algorithm for a standard linear mixed model for association analysis. In our simulations, we performed likelihood ratio tests in a univariate LMM for marker association mappings with a single phenotype. PLINK binary file format was [29] adopted as input files containing phenotypes and genetic information. A standardized relatedness matrix file was included to appropriately account for relatedness among subjects.

We applied GEMMA to analyze the simulated GWAS dataset and selected all SNPs with P-value below the threshold $5\times 10^{-6}$. Statistical power and type I error rate were calculated to evaluate the performance of marker association tests when the relatedness matrix used in LMMs was estimated by scGRM, UKin, or KING, respectively. The results suggest that all the methods have well controlled type I errors. We compared the power of association mapping and found the power of identifying risk variants was improved from 0.096 to 0.12 after we replaced scGRM with UKin in estimating pairwise kinship coefficients. For KING, the power was 0.04, which might be caused by the small SNP panel. This simulation suggests that the application of UKin can improve statistical power while controlling the type I error rate in GWAS. However, we failed to observe improvements in power in real data analyses, which suggests that the influence of different GRMs on association study is limited as only a small proportion of individual pairs from GWAS are related. Further simulations and real data experiments are required to evaluate the advantages of UKin over the scGRM and KING in association study comprehensively.

In this article, we have proved that there exist biases in different scGRM-based methods for estimating kinship relationship among individuals from genetic data. Beside genetic data, we may identify relationship or similarity among individuals using other kinds of data in real life. For example, we may want to infer the kinship among individuals based on human facial images, which is known as facial kinship verification problem in computer vision [30]. Or we may want to identify who is the speaker of an audio record based on recorded voices from different people, which is known as speaker identification problem [31]. In the machine learning community, metric learning and dictionary learning methods were proposed to tackle these two kinds of problems in general data setting [32, 33]. Metric learning methods [34] aim to automatically learn similarity from data. In dictionary learning [35], we intend to express the signal as the linear combination of different sources constituting a dictionary. Some supervised learning approaches, such as random forest and deep learning, can be adapted in the metric learning or dictionary learning scenarios [36–39]. In both scenarios, correlation is a major category of similarity measures [40, 41]. However, based on Property 3 we proved in the Additional file 1, there are potential biases for correlations since the sample means are used to center features. Further experiments are needed to evaluate how learning results may be impacted by such biases. Our debiased method, UKin, has potential to improve correlation-based metric learning and dictionary learning.

## Supplementary Information

**Additional file 1.** Technical details such as mathematical derivations and the results of simulations with 10,000 SNPs.

## Data Availability

The Young-Onset Breast Cancer Study can be found at dbGaP Study Accession: phs000678.v1.p1, while the Familial Intracranial Aneurysm Linkage Study can be found at dbGaP StudyAccession: phs000293.v1.p1. We make the source code for UKin calculation available at https://github.com/zxy22320/UKin.
