# Supplementary material for: An unbiased kinship estimation method for genetic data analysis
Source: BMC Bioinformatics. 2022 Dec 6;23:525. doi: 10.1186/s12859-022-05082-2 (PMC9727941; doi:10.1186/s12859-022-05082-2)
Supplement: Supplementary file 1 — Additional file 1. Technical details such as mathematical derivations and the results of simulations with 10,000 SNPs. [file 12859_2022_5082_MOESM1_ESM.pdf]

RESEARCH

An unbiased kinship estimation method for genetic data analysis

Wei Jiang[1†], Xiangyu Zhang[1†], Siting Li[2], Shuang Song[3,4] and Hongyu Zhao[1*]

*Correspondence: hongyu.zhao@yale.edu
[1]Department of Biostatistics, School of Public Health, Yale University, New Haven, United States of America
Full list of author information is available at the end of the article
†Equal contributor

Appendix

Proof of Property 1.

For the j-th single nucleotide polymorphism (SNP) $(1 \leq j \leq m)$, let f_j be the frequency of the reference allele (with label A) at that SNP. Consider a pair of individuals i and i' whose kinship coefficient is denoted by $\phi_{ii'}$, we derive the covariance of X_{ij} and $X_{i'j}$ from two different aspects. Recall that we denote $\rho_{ii',j}$ to be the correlation between X_{ij} and $X_{i'j}$, thus we have

$$Cov(X_{ij}, X_{i'j}) = \rho_{ii',j}\sigma_j^2. \tag{A.1}$$

On the other hand, X_{ij} can be treated as the sum of two independent Bernoulli random variables. That is, $X_{ij} = B_{ij(1)} + B_{ij(2)}$. For $k = 1, 2$,

$$B_{ij(k)} = \begin{cases} 0 & \text{if the } k \text{ th allele for } i \text{ at SNP } j \text{ is } a \\ 1 & \text{if the } k \text{ th allele for } i \text{ at SNP } j \text{ is } A \end{cases}.$$

With this expression of X_{ij} , we have

$$\begin{aligned} & Cov(X_{ij}, X_{i'j}) \\ = & Cov(B_{ij(1)} + B_{ij(2)}, B_{i'j(1)} + B_{i'j(2)}) \\ = & \sum_{k=1}^{2}\sum_{k'=1}^{2} Cov(B_{ij(k)}, B_{i'j(k')}) \\ = & \sum_{k=1}^{2}\sum_{k'=1}^{2} \{E[B_{ij(k)}B_{i'j(k')}] - E[B_{ij(k)}]E[B_{i'j(k')}]\}. \end{aligned} \tag{A.2}$$

As we denote f_j to be the probability that a random allele chosen from the j-th SNP is A. Notice that $B_{ij(k)}B_{i'j(k')} = 1$ only when the two alleles selected from i and i' at this marker are both with label A, under this circumstance, these two reference alleles are either identical by descent (IBD) or not. For simplicity, let $A_{ij}(k)$ represent the k-th alleles from individual i at SNP j, if we assume IBD genes have the same allelic types and non-IBD genes have independent allelic types, we

obtain

$$\begin{aligned}&\sum_{k=1}^{2}\sum_{k'=1}^{2} E[B_{ij(k)}B_{i'j(k')}]\\ =&\sum_{k=1}^{2}\sum_{k'=1}^{2}[P(A_{ij}(k)\ and\ A_{i'j}(k^{'})\ are\ IBD)f_j\\ &+(1-P(A_{ij}(k)\ and\ A_{i'j}(k^{'})\ are\ IBD))f_j^2].\end{aligned}$$

Consider the definitions of f_j and $\phi_{ii'}$, we obtain

$$E[B_{ij(k)}]E[B_{i'j(k')}] = f_j^2.$$

$$\phi_{ii'} = \frac{1}{4}\sum_{k=1}^{2}\sum_{k'=1}^{2} P(A_{ij}(k)\ and\ A_{i'j}(k^{'})\ are\ IBD).$$

Substituting them into (A.2), we get

$$Cov(X_{ij}, X_{i'j}) = 4\phi_{ii'} f_j(1-f_j). \tag{A.3}$$

As X_{ij} is the sum of two $i.i.d.$ Bernoulli random variables whose probability of success is f_j, we can derive that $\sigma_j^2 = 2f_j(1-f_j)$. Together with (A.1) and (A.3), we have

$$\rho_{ii',j} = 2\phi_{ii'}. \tag{A.4}$$

Equation (A.4) also reveals that the value of correlation $\rho_{ii',j}$ doesn't depend on which SNP is selected, thus we get

$$\begin{aligned}&\rho_{ii',1} = \rho_{ii',2} = \cdots = \rho_{ii',m} = \rho_{ii'}.\\ &\bar{\rho}_1 = \bar{\rho}_2 = \cdots = \bar{\rho}_m = \bar{\rho}.\end{aligned}$$

Proof of Property 2.

To demonstrate this property, we need a few preparations:

i. Consider the result $Cov(X_{ij}, X_{i'j}) = \rho_{ii'}\sigma_j^2$, we have

$$\begin{aligned}&EX_{ij}X_{i'j}\\ =&Cov(X_{ij}, X_{i'j}) + EX_{ij}EX_{i'j}\\ =&\rho_{ii'}\sigma_j^2 + \mu_j^2.\end{aligned} \tag{A.5}$$

Directly applying this result yields

$$
\begin{aligned}
&(1)EX_{ij}^2 = \sigma_j^2 + \mu_j^2. \\
&(2)E(\sum_{i=1}^{n} X_{ij}^2) = n(\sigma_j^2 + \mu_j^2). \\
&(3)E(\sum_{i=1}^{n}\sum_{i'<i} X_{ij}X_{i'j}) = \sum_{i=1}^{n}\sum_{i'<i}(\rho_{ii'}\sigma_j^2 + \mu_j^2) \\
&\quad = \frac{n(n-1)}{2}(\bar{\rho}\sigma_j^2 + \mu_j^2).
\end{aligned}
$$

ii. Based on (1)-(3) stated above, we have

$$
\begin{aligned}
&E\bar{X}_j^2 = E(\frac{1}{n}\sum_{i=1}^{n} X_{ij})^2 \\
&= \frac{1}{n^2}E(\sum_{i=1}^{n} X_{ij}^2 + 2\sum_{i=1}^{n}\sum_{i'<i} X_{ij}X_{i'j}) \\
&= \frac{n(\sigma_j^2 + \mu_j^2) + n(n-1)(\bar{\rho}\sigma_j^2 + \mu_j^2)}{n^2} \\
&= \frac{(n-1)\bar{\rho} + 1}{n}\sigma_j^2 + \mu_j^2. \qquad \text{(A.6)}
\end{aligned}
$$

With these preparations, we now work on the demonstration of Property 2.

Directly expand the expression on the left side of (A.2), we have

$$
\begin{aligned}
&E\left[\frac{(X_{ij} - \bar{X}_j)(X_{i'j} - \bar{X}_j)}{\sigma_j^2}\right] \\
&= \frac{1}{\sigma_j^2}[EX_{ij}X_{i'j} - EX_{ij}\bar{X}_j - EX_{i'j}\bar{X}_j + E(\bar{X}_j^2)].
\end{aligned}
$$

Substituting (A.5) and (A.6) into this expansion, we have

$$
\begin{aligned}
&E\Big[\frac{(X_{ij}-\bar{X}_j)(X_{i'j}-\bar{X}_j)}{\sigma_j^2}\Big] \\
&= \frac{1}{\sigma_j^2}[\rho_{ii'}\sigma_j^2+\mu_j^2-\frac{1}{n}(\sum_{\substack{a=1\\a\neq i}}^{n} EX_{ij}X_{aj}+EX_{ij}^2) \\
&\quad -\frac{1}{n}(\sum_{\substack{a=1\\a\neq i'}}^{n} EX_{i'j}X_{aj}+EX_{i'j}^2)+\frac{(n-1)\bar{\rho}+1}{n}\sigma_j^2+\mu_j^2] \\
&= \frac{1}{\sigma_j^2}[\rho_{ii'}\sigma_j^2+\mu_j^2-\frac{1}{n}(\sum_{\substack{a=1\\a\neq i}}^{n} \rho_{ia}\sigma_j^2+\sigma_j^2+n\mu_j^2) \\
&\quad -\frac{1}{n}(\sum_{\substack{a=1\\a\neq i'}}^{n} \rho_{ai'}\sigma_j^2+\sigma_j^2+n\mu_j^2)+\frac{(n-1)\bar{\rho}+1}{n}\sigma_j^2+\mu_j^2] \\
&= \frac{1}{\sigma_j^2}[\rho_{ii'}\sigma_j^2-\frac{1}{n}\sum_{\substack{a=1\\a\neq i}}^{n} \rho_{ia}\sigma_j^2-\frac{1}{n}\sigma_j^2-\frac{1}{n}\sum_{\substack{a=1\\a\neq i'}}^{n} \rho_{ai'}\sigma_j^2 \\
&\quad -\frac{1}{n}\sigma_j^2+\frac{(n-1)\bar{\rho}+1}{n}\sigma_j^2] \\
&= \rho_{ii'}-\frac{1}{n}\sum_{\substack{a=1\\a\neq i}}^{n} \rho_{ia}-\frac{1}{n}\sum_{\substack{a=1\\a\neq i'}}^{n} \rho_{ai'}+\frac{(n-1)}{n}\bar{\rho}-\frac{1}{n}. \qquad \text{(A.7)}
\end{aligned}
$$

Thus we derive the conclusion in Property 2.

Proof of Property 3.

For ease of calculation, we make a complement to the value range of index i':

$$
\begin{aligned}
&E\Big[\sum_{i=1}^{n}\sum_{i'=i+1}^{n}\frac{(X_{ij}-\bar{X}_j)(X_{i'j}-\bar{X}_j)}{\sigma_j^2}\Big] \\
&= \frac{1}{2}E\Big[\sum_{i=1}^{n}\sum_{i'\neq i}\frac{(X_{ij}-\bar{X}_j)(X_{i'j}-\bar{X}_j)}{\sigma_j^2}\Big] \\
&= \frac{1}{2}\sum_{i=1}^{n}\sum_{i'\neq i}E\Big[\frac{(X_{ij}-\bar{X}_j)(X_{i'j}-\bar{X}_j)}{\sigma_j^2}\Big]. \qquad \text{(A.8)}
\end{aligned}
$$

Equation(A.8) together with the conclusion (A.7) in the proof of Property 2 yields

$$
\begin{aligned}
&E\Big[\sum_{i=1}^{n}\sum_{i'=i+1}^{n}\frac{(X_{ij}-\bar{X}_j)(X_{i'j}-\bar{X}_j)}{\sigma_j^2}\Big] \\
&=\frac{1}{2}\sum_{i=1}^{n}\sum_{i'\neq i}[\rho_{ii'}-\frac{1}{n}\sum_{\substack{a=1\\a\neq i}}^{n}\rho_{ia}-\frac{1}{n}\sum_{\substack{a=1\\a\neq i'}}^{n}\rho_{ai'}+\frac{(n-1)\bar{\rho}}{n}-\frac{1}{n}] \\
&=\frac{n(n-1)}{2}\bar{\rho}-\frac{1}{2n}\sum_{i=1}^{n}\sum_{i'\neq i}\sum_{\substack{a=1\\a\neq i}}^{n}\rho_{ia}-\frac{1}{2n}\sum_{i=1}^{n}\sum_{i'\neq i}\sum_{\substack{a=1\\a\neq i'}}^{n}\rho_{ai'} \\
&\quad+\frac{n(n-1)^2}{2n}\bar{\rho}-\frac{n(n-1)}{2n} \\
&=\frac{(2n-1)(n-1)}{2}\bar{\rho}-\frac{1}{2n}\sum_{i=1}^{n}\sum_{i'\neq i}\sum_{\substack{a=1\\a\neq i}}^{n}\rho_{ia}-\frac{1}{2n}\sum_{i=1}^{n}\sum_{i'\neq i}\sum_{\substack{a=1\\a\neq i'}}^{n}\rho_{ai'} \\
&\quad-\frac{n(n-1)}{2n}.
\end{aligned}
$$

We observe that $\sum_{\substack{a=1\\a\neq i}}^{n}\rho_{ia}$ is irrelevant to i', therefore

$$
\begin{aligned}
&\frac{1}{2n}\sum_{i=1}^{n}\sum_{i'\neq i}\sum_{\substack{a=1\\a\neq i}}^{n}\rho_{ia} \\
&=\frac{n-1}{2n}\sum_{i=1}^{n}\sum_{\substack{a=1\\a\neq i}}^{n}\rho_{ia} \\
&=\frac{n-1}{2n}n(n-1)\bar{\rho}=\frac{(n-1)^2}{2}\bar{\rho}.
\end{aligned}
$$

Besides, if we change the sequence of summation, we have

$$
\begin{aligned}
&\frac{1}{2n}\sum_{i=1}^{n}\sum_{i'\neq i}\sum_{\substack{a=1\\a\neq i'}}^{n}\rho_{ai'} \\
&=\frac{1}{2n}\sum_{i'=1}^{n}\sum_{i\neq i'}\sum_{\substack{a=1\\a\neq i'}}^{n}\rho_{ai'} \\
&=\frac{n-1}{2n}n(n-1)\bar{\rho}=\frac{(n-1)^2}{2}\bar{\rho}.
\end{aligned}
$$

Substituting them into the expansion, we get

$$
\begin{aligned}
&E\Big[\sum_{i=1}^{n}\sum_{i'=i+1}^{n}\frac{(X_{ij}-\bar{X}_j)(X_{i'j}-\bar{X}_j)}{\sigma_j^2}\Big]\\
&=\frac{(2n-1)(n-1)}{2}\bar{\rho}-\frac{(n-1)^2}{2}\bar{\rho}-\frac{(n-1)^2}{2}\bar{\rho}-\frac{n(n-1)}{2n}\\
&=(\frac{(2n-1)(n-1)}{2}-(n-1)^2)\bar{\rho}-\frac{n-1}{2}\\
&=\frac{n-1}{2}\bar{\rho}-\frac{n-1}{2}=\frac{n-1}{2}(\bar{\rho}-1).
\end{aligned}
$$

Thus we finish the proof of Property 3.

Proof of Property 4.

At the start, we focus on a part of the expression on the left side:

$$
\begin{aligned}
&E\Big[\sum_{\substack{i'=1\\ i'\neq i}}^{n}\frac{(X_{ij}-\bar{X}_j)(X_{i'j}-\bar{X}_j)}{\sigma_j^2}\Big]\\
&=\sum_{\substack{i'=1\\ i'\neq i}}^{n}[\rho_{ii'}-\frac{1}{n}\sum_{\substack{a=1\\ a\neq i}}^{n}\rho_{ia}-\frac{1}{n}\sum_{\substack{a=1\\ a\neq i'}}^{n}\rho_{ai'}+\frac{(n-1)}{n}\bar{\rho}-\frac{1}{n}]\\
&=\sum_{\substack{i'=1\\ i'\neq i}}^{n}\rho_{ii'}-\frac{n-1}{n}\sum_{\substack{i'=1\\ i'\neq i}}^{n}\rho_{ii'}-\frac{1}{n}\sum_{\substack{i'=1\\ i'\neq i}}^{n}\sum_{\substack{a=1\\ a\neq i'}}^{n}\rho_{ai'}\\
&\quad+\frac{(n-1)^2}{n}\bar{\rho}-\frac{n-1}{n}\\
&=\frac{1}{n}\sum_{\substack{i'=1\\ i'\neq i}}^{n}\rho_{ii'}-\frac{1}{n}(\sum_{i'=1}^{n}\sum_{\substack{a=1\\ a\neq i'}}^{n}\rho_{ai'}-\sum_{\substack{a=1\\ a\neq i}}^{n}\rho_{ai})+\frac{(n-1)^2}{n}\bar{\rho}-\frac{n-1}{n}\\
&=\frac{2}{n}\sum_{\substack{i'=1\\ i'\neq i}}^{n}\rho_{ii'}-\frac{1}{n}n(n-1)\bar{\rho}+\frac{(n-1)^2}{n}\bar{\rho}-\frac{n-1}{n}\\
&=\frac{2}{n}\sum_{\substack{i'=1\\ i'\neq i}}^{n}\rho_{ii'}-\frac{n-1}{n}\bar{\rho}-\frac{n-1}{n}. \qquad \text{(A.9)}
\end{aligned}
$$

Substituting (A.7), together with (A.9), into the whole expansion, we get

$$
\begin{aligned}
&E\Big[\frac{(X_{ij}-\bar{X}_j)(X_{i'j}-\bar{X}_j)}{\sigma_j^2}+\frac{1}{2}\sum_{\substack{k=1\\k\neq i}}^{n}\frac{(X_{ij}-\bar{X}_j)(X_{kj}-\bar{X}_j)}{\sigma_j^2}\\
&\quad+\frac{1}{2}\sum_{\substack{l=1\\l\neq i'}}^{n}\frac{(X_{lj}-\bar{X}_j)(X_{i'j}-\bar{X}_j)}{\sigma_j^2}+1\Big]\\
&=\rho_{ii'}-\frac{1}{n}\sum_{\substack{a=1\\a\neq i}}^{n}\rho_{ia}-\frac{1}{n}\sum_{\substack{a=1\\a\neq i'}}^{n}\rho_{ai'}+\frac{(n-1)}{n}\bar{\rho}-\frac{1}{n}\\
&\quad+\frac{1}{2}(\frac{2}{n}\sum_{\substack{k=1\\k\neq i}}^{n}\rho_{ik}-\frac{n-1}{n}\bar{\rho}-\frac{n-1}{n})\\
&\quad+\frac{1}{2}(\frac{2}{n}\sum_{\substack{l=1\\l\neq i'}}^{n}\rho_{li'}-\frac{n-1}{n}\bar{\rho}-\frac{n-1}{n})+1\\
&=\rho_{ii'}-\frac{1}{n}-\frac{n-1}{n}+1\\
&=\rho_{ii'}.
\end{aligned}
$$

Here we have proved the conclusion in Property 4.

robust GRM and two-step GRM

A general class of GRM estimators have the following form:

$$\hat{\rho}_{ii'}=\sum_{j=1}^{m}w_j\times\frac{X_{ij}X_{i'j}-a_j(X_{ij}+X_{i'j})+4a_jp_j-4p_j^2}{2p_j(1-p_j)},$$

where $p=(p_1,...,p_m)^T$ are the population frequencies of the reference alleles , $a=(a_1,...,a_m)^T$ are multiplicative factors, and $w=(w_1,...,w_m)^T$ are non-negative weights satisfying $\sum_{j=1}^{m}w_j=1$.

The sample version is:

$$\hat{\rho}_{ii'}=\sum_{j=1}^{m}w_j\times\frac{X_{ij}X_{i'j}-a_j(X_{ij}+X_{i'j})+2a_j\bar{X}_j-\bar{X}_j^2}{\sigma_j^2},$$

where $\sigma_j^2=2p_j(1-p_j)=Var(X_{ij})$, which can be replaced by a consistent estimator $\hat{\sigma}_j^2$ in practice.

The scGRM estimator is a special case with $a_j=\bar{X}_j$ and $w_j=1/m$ for $j=1,...,m$, which has been proved to be biased.

The robust GRM estimator is a special case with $a_j = \bar{X}_j$ and $w_j = \sigma_j^2/(\sum_{l=1}^m \sigma_l^2)$ for all j, i.e.

$$\begin{aligned}\hat{\rho}_{ii'}^r =& \sum_{j=1}^m \frac{\sigma_j^2}{\sum_{l=1}^m \sigma_l^2} \times \frac{(X_{ij}-\bar{X}_j)(X_{i'j}-\bar{X}_j)}{\sigma_j^2}\\ =& \frac{\sum_{j=1}^m (X_{ij}-\bar{X}_j)(X_{i'j}-\bar{X}_j)}{\sum_{l=1}^m \sigma_l^2}.\end{aligned}$$

Therefore, with Property 2 in the method section, we have

$$\begin{aligned}E[\hat{\rho}_{ii'}^r] =& \sum_{j=1}^m \frac{\sigma_j^2}{\sum_{l=1}^m \sigma_l^2} \times E\{\frac{(X_{ij}-\bar{X}_j)(X_{i'j}-\bar{X}_j)}{\sigma_j^2}\}\\ =& \sum_{j=1}^m \frac{\sigma_j^2}{\sum_{l=1}^m \sigma_l^2}\{\rho_{ii'} - \frac{1}{n}\sum_{\substack{a=1\\ a\neq i}}^n \rho_{ia} - \frac{1}{n}\sum_{\substack{a=1\\ a\neq i'}}^n \rho_{ai'} + \frac{(n-1)}{n}\bar{\rho} - \frac{1}{n}\}\\ =& \rho_{ii'} - \frac{1}{n}\sum_{\substack{a=1\\ a\neq i}}^n \rho_{ia} - \frac{1}{n}\sum_{\substack{a=1\\ a\neq i'}}^n \rho_{ai'} + \frac{(n-1)}{n}\bar{\rho} - \frac{1}{n}.\end{aligned}$$

Therefore, rGRM estimator is still biased.

The global Day-Williams estimator is a special case with $a_j = 1$ and $w_j = \sigma_j^2/(\sum_{l=1}^m \sigma_l^2)$ for all j, i.e

$$\hat{\rho}_{ii'}^d = \frac{\sum_{j=1}^m\{X_{ij}X_{i'j} - (X_{ij}+X_{i'j}) + 2\bar{X}_j - \bar{X}_j^2\}}{\sum_{l=1}^m \sigma_l^2}.$$

From the proof of property 2, we know

$$EX_{ij}X_{i'j} = \rho_{ii'}\sigma_j^2 + \mu_j^2, \quad E\bar{X}_j^2 = \frac{(n-1)\bar{\rho}+1}{n}\sigma_j^2 + \mu_j^2.$$

Therefore,

$$\begin{aligned}E\hat{\rho}_{ii'}^d =& \frac{\sum_{j=1}^m\{E[X_{ij}X_{i'j}] - EX_{ij} - EX_{i'j} + 2E\bar{X}_j - E\bar{X}_j^2\}}{\sum_{l=1}^m \sigma_l^2}\\ =& \frac{\sum_{j=1}^m\{\rho_{ii'}\sigma_j^2 + \mu_j^2 - 2\mu_j + 2\mu_j - \frac{(n-1)\bar{\rho}+1}{n}\sigma_j^2 - \mu_j^2\}}{\sum_{l=1}^m \sigma_l^2}\\ =& \frac{\sum_{j=1}^m(\rho_{ii'} - \frac{(n-1)\bar{\rho}+1}{n})\sigma_j^2}{\sum_{l=1}^m \sigma_l^2}\\ =& \rho_{ii'} - \frac{(n-1)\bar{\rho}+1}{n}.\end{aligned}$$

Therefore, the global Day-Williams estimator still has a negative bias.

Write

$$Z_j(a_j) = \frac{X_{ij}X_{i'j} - a_j(X_{ij}+X_{i'j}) + 4a_jp_j - 4p_j^2}{\sigma_j^2},$$

so that $\hat{\rho}_{ii'} = \sum_{j=1}^{m} w_j \times Z_j(a_j)$. Then under linkage equilibrium, we have

$$Var(\hat{\rho}_{ii'}) = \sum_{j=1}^{m} w_j^2 V_j(a_j),$$

where $V_j(a_j) = Var[Z_j(a_j)]$. The author suggest one should first choose a_j that minimizes $V_j(a_j)$, and then choose w minimize $Var(\hat{\rho}_{ii'})$, which results in

$$\hat{a}_j = \frac{1}{1+\rho_{ii'}} \times 2p_j + \frac{\rho_{ii'}}{1+\rho_{ii'}}, \quad \hat{w}_j = \frac{V_j(a_j)^{-1}}{\sum_{l=1}^{m} V_l(a_l)^{-1}}.$$

The two-step GRM estimator is then defined as $\hat{\rho}_{ii'}^{T} = \sum_{j=1}^{m} \hat{w}_j \times Z_j(\hat{a}_j)$.

Under the sample version:

$$\begin{aligned}
EZ_j(\hat{a}_j) =& E\{\frac{X_{ij}X_{i'j} - \hat{a}_j(X_{ij} + X_{i'j}) + 2\hat{a}_j\bar{X}_j - \bar{X}_j^2}{\sigma_j^2}\} \\
=& E\{\frac{\frac{1}{1+\rho_{ii'}}(X_{ij} - \bar{X}_j)(X_{i'j} - \bar{X}_j) + \frac{\rho_{ii'}}{1+\rho_{ii'}}(X_{ij}X_{i'j} - (X_{ij} + X_{i'j}) + 2\bar{X}_j - \bar{X}_j^2)}{\sigma_j^2}\} \\
=& \frac{1}{1+\rho_{ii'}}(\rho_{ii'} - \frac{1}{n}\sum_{\substack{a=1 \\ a \neq i}}^{n} \rho_{ia} - \frac{1}{n}\sum_{\substack{a=1 \\ a \neq i'}}^{n} \rho_{ai'} + \frac{(n-1)}{n}\bar{\rho} - \frac{1}{n}) \\
&+ \frac{\rho_{ii'}}{1+\rho_{ii'}}(\rho_{ii'} - \frac{(n-1)\bar{\rho}+1}{n}) \\
=& \rho_{ii'} + \frac{1-\rho_{ii'}}{1+\rho_{ii'}}\frac{(n-1)}{n}\bar{\rho} - \frac{1}{n} - \frac{1}{1+\rho_{ii'}}(\frac{1}{n}\sum_{\substack{a=1 \\ a \neq i}}^{n} \rho_{ia} + \frac{1}{n}\sum_{\substack{a=1 \\ a \neq i'}}^{n} \rho_{ai'}).
\end{aligned}$$

Because $EZ_j(\hat{a}_j)$ doesn't depend on j, thus no matter how we choose $\hat{w}$,

$$E\hat{\rho}_{ii'}^{T} = \sum_{j=1}^{m} \hat{w}_j \times EZ_j(\hat{a}_j) = \rho_{ii'} + \frac{1-\rho_{ii'}}{1+\rho_{ii'}}\frac{(n-1)}{n}\bar{\rho} - \frac{1}{1+\rho_{ii'}}(\frac{1}{n}\sum_{\substack{a=1 \\ a \neq i}}^{n} \rho_{ia} + \frac{1}{n}\sum_{\substack{a=1 \\ a \neq i'}}^{n} \rho_{ai'}) - \frac{1}{n},$$

which indicates the two-step GRM estimator is still biased.

Table A1: Comparison of UKin, KING and scGRM in biases and SDs (10,000 SNPs).

True Value	Bias from True Value ($\times 10^{-3}$) (UKin)	(KING)	(scGRM)	Standard deviation ($\times 10^{-3}$) (UKin)	(KING)	(scGRM)
0.000	**-0.368**	−2.079	−0.899	3.050	4.247	**2.261**
0.125	**-0.540**	−1.699	−0.783	**2.230**	3.204	2.616
0.250	**-0.599**	−1.245	−1.244	**2.335**	2.658	2.646
0.500	**0.000**	**0.000**	−1.218	**0.000**	**0.000**	3.251

Author details

[1]Department of Biostatistics, School of Public Health, Yale University, New Haven, United States of America.
[2]Department of Biomedical Data Science, Geisel School of Medicine, Dartmouth College, Hanover, United States of America. [3]Center for Statistical Science, Tsinghua University, Beijing, China. [4]Department of Industrial Engineering, Tsinghua University, Beijing, China.
